# A mixed‐methods study of the management of hearing loss associated with otitis media with effusion in children with Down syndrome

**DOI:** 10.1111/coa.13228

**Published:** 2018-10-08

**Authors:** Amanda Hall, Helen Pryce, Iain A. Bruce, Peter Callery, Monica Lakhanpaul, Anne G. M. Schilder

**Affiliations:** ^1^ Children's Hearing Centre St Michael's Hospital University Hospitals Bristol NHS Foundation Trust Bristol UK; ^2^ Life and Health Sciences Aston University Birmingham UK; ^3^ Paediatric ENT Department Royal Manchester Children's Hospital Manchester University NHS Foundation Trust Manchester Academic Health Science Centre Manchester UK; ^4^ Division of Infection, Immunity and Respiratory Medicine Faculty of Biology, Medicine and Health University of Manchester Manchester UK; ^5^ School of Health Sciences University of Manchester Manchester UK; ^6^ UCL Great Ormond Street Institute of Child Health London UK; ^7^ Community Paediatrics Whittington Health NHS London UK; ^8^ evidENT, Ear Institute University College London London UK

**Keywords:** child, community participation, Down syndrome, hearing aids, middle ear ventilation, otitis media, otolaryngology

## Abstract

**Objectives:**

To scope current service provision across England for management of otitis media with effusion and hearing loss in children with Down syndrome; to explore professional decision‐making about managing otitis media with effusion and hearing loss; and to explore patient and public views on the direction of future research.

**Design:**

Mixed methods including a service evaluation of NHS clinical practice through a structured telephone survey; a qualitative study of professional decision‐making with in‐depth interviews collected and analysed using grounded theory methods; patient/public involvement consultations.

**Participants:**

Twenty‐one audiology services in England took part in the evaluation; 10 professionals participated in the qualitative study; 21 family members, 10 adults with Down syndrome and representatives from two charities contributed to the consultations.

**Results:**

There was variation across services in the frequency of routine hearing surveillance, approaches to managing conductive hearing loss in infancy and provision of hearing aids and grommets. There was variation in how professionals describe their decision‐making, reflecting individual treatment preferences, differing approaches to professional remit and institutional factors. The consultations identified that research should focus on improving practical support for managing the condition and supporting decision‐making about interventions.

**Conclusions:**

There is system‐level variation in the provision of services and individual‐level variation in how professionals make clinical decisions. As a consequence, there is inequity of access to hearing health care for children with Down syndrome. Future research should focus on developing core outcomes for research and care, and on improving decision support for families.

1

**Keypoints**

- Otitis media with effusion and hearing loss is common in children with Down syndrome and contributes to difficulties in listening and communication.
- The common interventions for otitis media with effusion and hearing loss have associated risks specific to children with Down syndrome and there is little evidence to guide clinical management for this group of children.
- It is not known how services are delivered or professionals make decisions about interventions.
- There is system level variation in the provision of services for children with Down syndrome affected by otitis media with effusion and hearing loss, as well as individual level variation in how professionals make clinical decisions.
- Future research should focus on developing core outcomes for research and care, and on improving decision support for children with Down syndrome and their families.

## 2 INTRODUCTION

Otitis media with effusion (OME) and its associated hearing loss are particularly prevalent in children with Down syndrome (DS).^1^ Early persistent hearing loss in this vulnerable group of children contributes to difficulties in listening, communication, behaviour and learning.^1^, ^2^ Intervention options for hearing loss associated with OME include ventilation tubes (grommets), hearing aids (air and bone conduction) and conservative observation (watchful waiting). There is little evidence regarding the effectiveness of these management strategies in children with DS, with each having associated risks specific to children with DS. For example, grommet surgery may be difficult due to a narrow ear canal and children with DS are at increased risk of grommet associated ear discharge and eardrum perforation.^3^ Air conduction hearing aids often do not fit well or exacerbate ear infections. Consequently, there is a need for further research regarding the effectiveness, acceptability and utilisation of management strategies for hearing loss associated with persistent OME, in children and young people with DS.

A recent project examined the feasibility and value of conducting research on management of OME in children with DS^1^: key findings were that parents had experienced a range of treatments and interventions for OME (as above), and perceive that management of their child's OME and hearing loss is inconsistent and based on uncertain foundations.^1^ The top three interventions rated by clinicians to be most effective in children with DS were conventional hearing aids, bone‐anchored auditory devices (softband or implanted) and grommets,^1^ but the study did not investigate how professionals make decisions about these interventions or the preferred and actual patient pathway.

The objectives of this study are, for children with DS, OME and hearing loss, to:

1. Scope the range of current service provision across England;
2. Explore professional decision‐making; and
3. Explore patient, parent and public views on the direction of future research.

## 3 METHODS

This was a mixed**‐**methods study involving a service evaluation of NHS clinical practice for managing OME and hearing loss in children with DS and a qualitative investigation using grounded theory methods of professional decision‐making in this patient group. Alongside, we ran patient, parent and public involvement groups to explore future research topics.

### 3.1 Service evaluation

A telephone survey was conducted with clinicians in England who had responsibility for hearing services for children with DS. Clinicians were recruited through professional contacts as well as through a short online questionnaire to members of the Down Syndrome Medical Interest Group and British Academy of Audiology asking about the range of interventions for OME and hearing loss offered to children with DS in their department (air‐bone conduction hearing aids, bone‐anchored auditory devices on softbands, grommets) and willingness to take part in a 30‐minute telephone survey. Services were sampled to include those providing bone‐anchored auditory devices and air conduction hearing aids. The survey was structured to cover: protocols for hearing surveillance, criteria for intervention, types of interventions provided, outcome measures, the level of multidisciplinary involvement and parent involvement in decision‐making in their service.

### 3.2 Qualitative study of professional decision‐making

We used grounded theory methods^4^ to theorise the process by which professionals from different professional groups make decisions about managing hearing loss in children with DS. Professionals who work with children with DS and hearing loss were sent an invitation to participate in the study through regional audiology, ENT and child health networks and clinical contacts. The researcher initially interviewed those who responded first, and then selected from those who responded to provide contrast in profession, caseload and clinical population.^4^ Professionals sampled included hearing support teachers, community paediatricians, audiologists and ENT surgeons. An optometrist was also sampled as it became apparent that this professional group is involved in decision‐making about hearing aids in relation to eyeglasses. Volunteer participants contacted the researcher directly, and arrangements were made to conduct face‐to‐face interviews in the workplace where possible, or phone interviews.

The researcher (AH), who is also an audiologist, conducted in‐depth interviews guided by a topic guide (Table 1). They lasted between 30 and 60 minutes and were audio‐recorded, transcribed verbatim and anonymised. Using an iterative process, data were gathered and analysed concurrently using the constant comparison method of grounded theory. Interviews were analysed using open and axial coding techniques; each statement was allocated a code and codes were linked from each data source into themes with variation in properties and dimensions. Reflective memos were kept during the process of data collection and analysis, and these were used to check emerging themes. Themes were grouped into a framework of decision‐making. Transcripts were read by a second researcher (HP), an experienced qualitative researcher, who blind‐coded a selection of data and checked derived codes.

**Table 1:** Topic guide

The nature of the participant's professional experience of children with DS
Experience of the nature of OME and hearing problems in children with DS
Opinions and experience of different interventions for managing OME and hearing loss in children with DS
How participants make decisions about managing OME and hearing loss in children with DS
Participants views on the barriers and facilitators to decision‐making about OME and hearing loss in children with DS
Participants’ views on parental involvement in decision‐making for children with DS
Participants views on parental experiences and expectations of managing OME and hearing loss in children with DS

### 3.3 Patient and public involvement

Over the course of the study, PPI groups were held. These involved parents and carers of children with DS, people with DS, a charitable organisation representing people with DS and a charitable organisation representing children with hearing loss. The aim of the groups was to identify research topics and priorities relating to OME and hearing loss that are important to them as service users, to present the topics arising from the research and to discuss future research priorities. The findings which arose are grouped according to common topics and a narrative summary presented.

### 3.4 Ethical considerations

The service evaluation was conducted with agreement from University Hospitals Bristol NHS Foundation Trust Research and Innovation department (2015). R&D approval and NHS Permission for Research were obtained for the qualitative study through the University Hospitals Bristol NHS Trust (16/6/2015) and informed, written consent was obtained from all research participants. In line with National Institute of Healthcare Research guidance,^5^ ethical approval was not required for the PPI consultations as those involved were planning future research rather than participating in current research.

## 4 RESULTS

Data were obtained on the service provision for children with DS of 21 audiology services across the South West, London, East Midlands, Yorkshire and Humber, North East, South East and East of England.

Ten professionals working with children with DS and hearing loss, covering a range of professional groups, took part in qualitative interviews. The participants worked in, or with, five different NHS services in England. Due to the small sample size, no further details are given to protect their anonymity.

Three PPI groups were held and attended by a total of twelve mothers and one grandmother; additionally, eight parents provided feedback outside the groups. One group was held and attended by ten adults with DS. Consultation with representatives from two charities also took place.

### 4.1 Variation in service delivery

There was variation in key aspects of service delivery for children with DS (Table 2). All services report that parents are involved in decision‐making and are provided with written information.

**Table 2:** Summary of the main variation in hearing service provision for children with DS

Frequency of routine hearing tests	All services follow Down Syndrome Medical Interest Group hearing surveillance guidelines^6^ as a minimum Services adapt the guidelines with some offering more frequent tests, particularly in pre‐school years There is a twofold difference in the number of routine tests offered across services
Approaches to managing conductive hearing loss in infancy	Two main approaches: ○ Hearing loss is managed in infancy with hearing aids ○ No intervention in infancy; hearing loss is monitored and treated later if persistent
Provision of hearing aids	Hearing aids are the preferred intervention for managing OME‐related conductive hearing loss in childhood A range of approaches used: ○ Primarily air conduction hearing aids ○ Primarily bone conduction hearing aids ○ Parental choice determines hearing aid type Cost is a factor limiting use of bone‐anchored auditory devices on a softband for some services
Provision of grommets for treating OME‐related conductive hearing loss	Two main approaches: ○ Grommets rarely used ○ Grommets rarely used in early childhood but may be provided when child is older
Professional responsibility	Two main approaches. Management and decision‐making for OME and hearing loss led by either: ○ Audiology ○ ENT
Involvement of education hearing support services	Support from education hearing support services for children with OME and hearing loss varies: ○ No support ○ No routine support ○ Support only if hearing aids are worn ○ Support only for pre‐school children with hearing aids

### 4.2 Professional views on decision‐making

**Figure 1:**
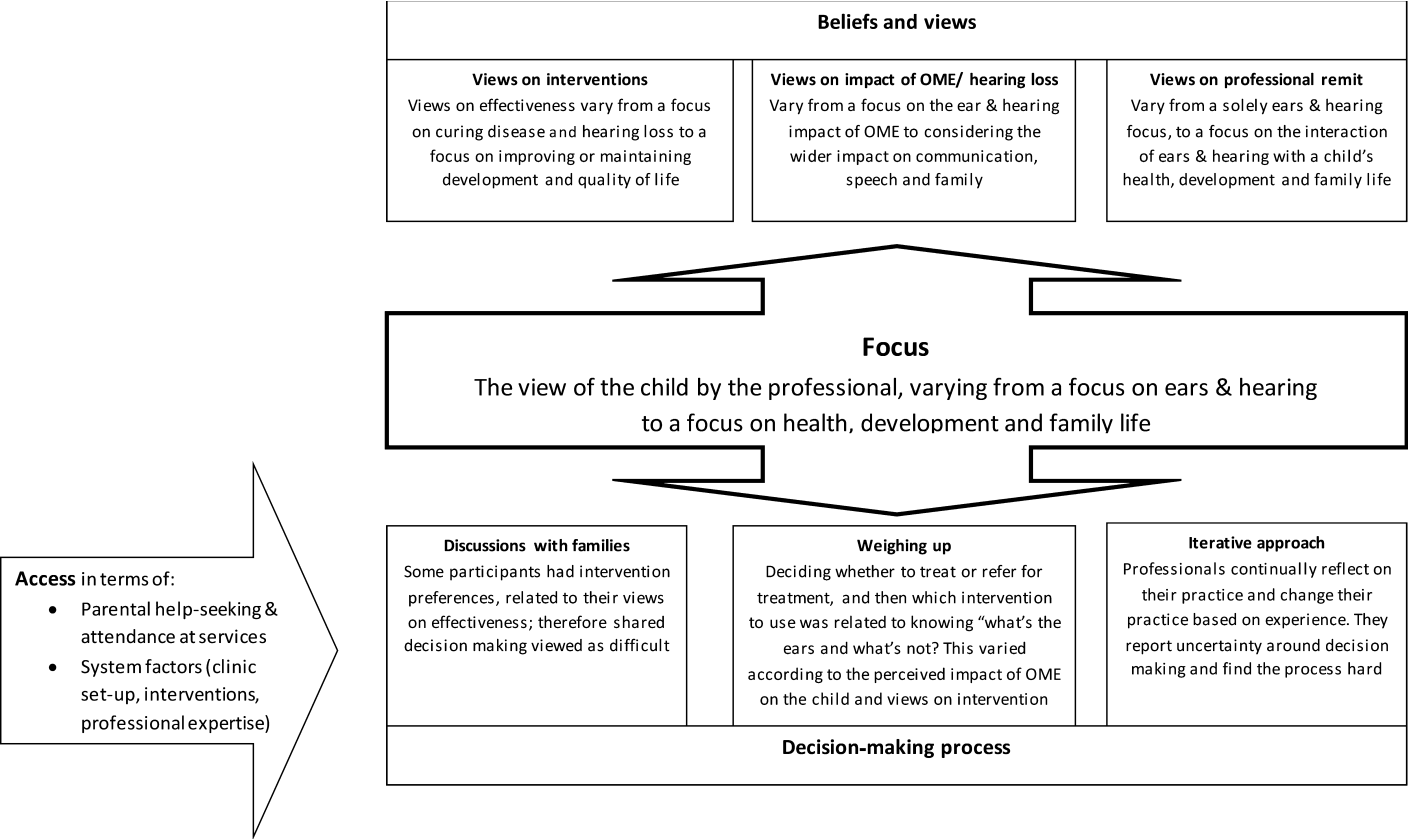
Framework of professional decision‐making

There was variation in how professionals describe their decision‐making about OME and hearing loss in children with DS. These variations reflect individual preferences, differing approaches to professional remit and institutional factors. The key theme, labelled “focus,” refers to how the child's life is viewed by the professional and whether their focus is primarily on a child's ears and hearing or on their health, development, family and school life. This is influenced by how they viewed their professional remit, how the relative burdens and benefits of interventions and treatments were perceived and ultimately how decision‐making was described (Figure 1). Key themes are described below, with example quotations in Table 3.

**Table 3:** Example quotes relating to the key themes (Figure 1 illustrates how the themes link)

Theme	Professional's quote
Views on professional remit—differing focus within professions	“You could say I need to be better informed about the medical side of it, and the implications of things like Down syndrome in a general way but …I think well that's not..my job” [*Paediatric audiologist 1*] “Making sure that if you said a family need help with toilet training, then actually that happens” [*Paediatric audiologist 2*]
Views on impact of OME and hearing loss showing differing focus	“big holes [in the ear drums] on both sides or they've got substantial hearing loss with those” [*ENT surgeon*] “hearing's huge isn't it? You know if you can't hear it's so hard to communicate with the children …having a child who can't hear changes how a family can run” [*Community paediatrician*]
Approach to treatment influenced by ear vs child focus	“What's important is that they get used to the idea of something on their head quite easily, quite early because once you've missed a certain window they'll rip it off” [*ENT surgeon*] “We don't really know what the child's potential is in terms of spoken language and understanding, so they should be having sign classes from the word go..” [*Paediatric audiologist*]
Professionals have preferences for treatment	“I suppose we don't push hearing aids a lot. I don't anyway” [*Audiology paediatrician*] “I suppose for me I just think, general anaesthetic..or a piece of equipment that you can take.. so that's why I'm biased towards [hearing aids]” [*Paediatric audiologist*] “Well I suppose I would present all of the options along with the pros and cons…. I don't think you can say what's going to be best for one family or another” [*Community paediatrician*]
System factors influencing access to interventions	“They [audiology services] just don't have the funding to dish out BAHAs or mini contacts for that matter” *[ENT surgeon]* “If it's purely conductive [hearing loss] we don't have access to teachers of the deaf.” [*Audiology Paediatrician*]
Weighing up decisional processes—ears or not?	“how can you know if it's a Down syndrome issue if they've got a 40 dB hearing loss and you haven't done anything to correct that” [*Paediatric audiologist*] “But he was not challenged enough to behave, so in the mainstream classroom he was really difficult, so I'd go in, and you sort of think, aah do you know what, your ears are not what's really the issue here” [*Hearing support teacher*]
Shared decision‐making—related to views on interventions	“you're giving parents choice but actually in my professional opinion, without hearing aids this child is not going to hear what they need to acquire speech, so I'm a bit more foisting that on them” [*Paediatric audiologist*]

#### 4.2.1 Professional remit

Views on professional remit varied from those who viewed ears and hearing loss alone as being within their remit, to those who viewed their remit as encompassing the interaction of hearing loss with a child's health and development. Although views on remit were related to the professional role of the participant with Audiologists and ENT surgeons more likely to have an ear and hearing focus, variation in viewpoint was also observed within participants of the same professional group.

#### 4.2.2 Impact of OME and hearing loss

There was variation in how the impact of OME and hearing loss on a child with DS was viewed. Some participants primarily focused on the ear and hearing impact of the condition, whereas others considered the impact on speech and communication and the wider impact on the child and family. Those who focused on ears and hearing described decision‐making as primarily focused on alleviating the OME or hearing loss, viewing hearing aids or surgery as the solution. This contrasted with those who took an approach to decision‐making focused on developing listening, communication and participation. Views on treatment and intervention success varied from a focus on curing OME or wearing hearing aids to those who viewed success as improving or maintaining quality of life or developmental outcomes, not always involving hearing aids or surgery and including sign language and other communication tactics.

#### 4.2.3 Professionals have treatment and intervention preferences

There was evidence of preferences amongst many of the professional participants for particular treatments and interventions for children with DS, with some preferring grommets and others hearing aids. This was not related to professional group, and there was variation in preference within groups. Participants described observing colleagues having preferences. A smaller number of participants did not identify a preference and described how they present the pros and cons about different options to families.

#### 4.2.4 Decision‐making processes

Many of the participants described the difficulty of being able to differentiate between the impact of hearing loss and the inherent learning disabilities of children with DS, of knowing “what's the ears and what's not?” The approach to this issue varied according to the focus on the child with DS. For those whose focus extended beyond ears and hearing, determining the impact of the hearing loss on the child was fundamental to decision‐making. Decision‐making involved, for example, deciding whether a child's behavioural difficulties were the result of hearing loss or a characteristic of DS. Decision to refer for treatment or to treat was made when hearing loss was felt to be having a negative impact on the child. For those participants who primarily focused on ears and hearing, understanding the impact on the child with DS was less important. Decision‐making for these participants was made primarily on diagnostic ear and hearing information, with hearing aids and grommets more likely to be prescribed. Those who considered other aspects of the child, such as their sensory sensitivity or likely tolerance, were more nuanced in their discussion around decision‐making.

#### 4.2.5 Context

The context in which decisions were made was influenced by availability of professional expertise, access to treatments and interventions, the health service set‐up and the child's educational setting. Having highly skilled professionals within a service provided a context in which decisions could be made effectively, such as having access to surgical expertise in children with DS and therefore ability to refer for grommet intervention or audiologists with experience of working with a range of hearing aids.

### 4.3 Parent, patient and public involvement

Parents discussed their ideas for future research through relating their personal experiences, both positive and negative. Parents were asked to discuss their views on the importance of future research on improving decision support and improving service provision and management of OME and hearing loss. Adults with DS attended a group workshop on the topic of ears and hearing; they discussed their personal experiences and knowledge about hearing, hearing loss and audiology services. Charity representatives gave their perspectives (summarised in Table 4).

**Table 4:** Research areas for OME and hearing loss in children with DS as identified by people with DS, family members and charity representatives

Research area	
Decision support	Improve support for parents making decisions about interventions Improve information about interventions for OME and hearing loss including the risks, benefits and likely outcomes
Support for parents and children with DS for managing hearing loss and OME	Increase awareness and improve practical support for managing hearing loss, with consideration for children wearing both hearing aids and glasses Develop information and materials for children and young people with DS about tests, procedures and interventions Improve wax management including development of less painful methods for removing wax Improve support for hypersensitivity to sound Develop tools for parents to detect hearing loss, monitor their child's hearing and assess whether hearing aids are of benefit
Support in school	Improve support for children with OME and hearing loss in school Increase awareness of hearing loss in school
Hearing, speech, communication and development	Understand how to optimise speech and communication in children with hearing loss Understand how to optimise hearing and learning to support a child's development Develop hearing and communication tactics training for parents and children
Health services	Improve integrated care, particularly between hearing and speech services Improve parental confidence in the hearing assessment process Improve access to the full range of hearing interventions, particularly bone‐anchored auditory devices on a softband

## 5 DISCUSSION

### 5.1 Synopsis of key findings

Using mixed methods and involving a range of professionals as well as people with DS and families, we have identified that across England there is system‐level variation in the provision of services for children with DS affected by OME and hearing loss, as well as individual‐level variation in how professionals make clinical decisions. As a consequence, there is inequity of access to health care for children with DS, OME and hearing loss. Parents of children with DS, people with DS and representatives of charitable organisations in DS describe variation in their experiences of care and believe that future research should focus on improving decision support, and developing improved information and support for managing newly diagnosed and ongoing OME and related issues.

### 5.2 Strengths of the study

Our approach enabled us to study the hearing services provided to children with DS from a range of perspectives, including that of the “system,” professionals and service users. The strong engagement with a range of stakeholders is a key strength of our study and has laid the foundations for future collaborative research. The sampling method used for our service evaluation enabled us to scope the range of approaches used, although we may have missed some alternative approaches and the results may not be generalisable to all of the UK. The qualitative study included in‐depth interviews about clinical decision‐making in children with DS with ten professionals representing different health and educational specialities. Key themes were identified, but the data were not fully saturated, and a full grounded theory could not be developed. Future work should include parents and people with DS to explore decision‐making from their perspectives. People with DS, parents and public were widely involved, and their viewpoints on the direction of future research were included.

### 5.3 Comparison with other studies

In line with the model of candidacy for health care described by Dixon‐Woods et al,^7^ access to services and management of OME and hearing loss in children with DS is related to professionals’ adjudication of candidacy for hearing interventions, which our data show is influenced by professionals having treatment and intervention preferences. Management of OME and hearing loss can be regarded as preference‐sensitive care; in that, there is not one optimal intervention or treatment, but there are trade‐offs to make to decide on the best treatment.^8^ For children without DS, taking no action is also a reasonable option, but this may not be the case for children with DS. Unlike for typically developing children, there is evidence that OME and hearing loss have an impact on language development in children with DS,^2^ but research is lacking on other developmental outcomes and effectiveness of interventions. This lack of evidence may make it difficult for clinicians to know what is best for children with DS, and they may rely on guidance developed for children without DS. With patient‐centred care now established as a core component of NHS care, trade‐offs and decision‐making about interventions should involve parents and children and not be based on clinician preferences. This can be achieved through implementing shared decision‐making (SDM) to enable parents and young people to make fully informed choices about interventions and treatment. To do this, parents and professionals need ready access to information, and professionals need guidance and tools on sharing decisions and discussing treatment pros and cons, as well as a clinical culture amenable to SDM.^9^ There are currently no suitable SDM tools available for OME and hearing loss in children with DS, in part due to lack of evidence to inform discussions and decisions, particularly around hearing aid provision. There is a necessity for research that accounts for the range of needs of children with DS, the trade‐offs and the complexity of managing OME and hearing loss and seeks to determine what works, for whom, under what circumstances and how.^10^

### 5.4 Clinical applicability

System constraints impact on adjudication of candidacy. These constraints were described in our data, such as availability of local clinical expertise, and these influenced treatment decisions. Given the lack of accepted national guidance about the management of OME and hearing loss in children with DS, practice is often decided at a local level and we observed variation in how services are delivered. The NICE guideline on surgical management of OME for children^11^ does not specify treatment criteria for children with DS, despite them being both at higher risk for OME and likely to be more adversely affected by its associated hearing loss. This may be due to the lack of evidence in children with DS and lack of consensus amongst professionals, and likely contributes to inequalities and unwarranted variations in care.^12^

Based upon these findings, further work for children with DS affected by OME and hearing loss should include the following: (a) developing tools for SDM and supporting parents in managing the condition; (b) deciding on the core outcomes for future research; (c) deciding on the key patient, clinical and quality outcomes for clinical services and commissioners; and (d) developing evidence: what works for which children with DS, to inform future management guidelines.

## CONFLICT OF INTEREST

All authors have completed the conflict of interest disclosure form and declare AS was previously supported through an NIHR Research Professorship at UCL and is now supported in part by the NIHR University College London Hospitals Biomedical Research Centre; no financial relationships with any organisations that might have an interest in the submitted work in the previous three years; and no other relationships or activities that could appear to have influenced the submitted work.
